# The role of CXCL10 in the pathogenesis of experimental septic shock

**DOI:** 10.1186/cc13902

**Published:** 2014-06-02

**Authors:** Daniela S Herzig, Liming Luan, Julia K Bohannon, Tracy E Toliver-Kinsky, Yin Guo, Edward R Sherwood

**Affiliations:** 1 Department of Anesthesiology, The University of Texas Medical Branch and Shriners Hospital for Children, Galveston, Texas, USA; 2 Department of Biochemistry and Molecular Biology, The University of Texas Medical Branch, Galveston, Texas, USA; 3 Department of Anesthesiology, Vanderbilt University Medical Center, Nashville, Tennessee, USA; 4 Department of Pathology, Microbiology and Immunology, Vanderbilt University Medical Center, Nashville, Tennessee, USA; 5 Department of Obstetrics and Gynecology-Großhadern, Ludwig-Maximilians-University, Munich, Germany; 6 Vanderbilt University Medical Center Anesthesiology Research Division, 1161 21st Avenue South, T-4202 MCN, Nashville, TN 37232-2520, USA

## Abstract

**Introduction:**

The chemokine CXCL10 is produced during infection and inflammation to activate the chemokine receptor CXCR3, an important regulator of lymphocyte trafficking and activation. The goal of this study was to assess the contributions of CXCL10 to the pathogenesis of experimental septic shock in mice.

**Methods:**

Septic shock was induced by cecal ligation and puncture (CLP) in mice resuscitated with lactated Ringer’s solution and, in some cases, the broad spectrum antibiotic Primaxin. Studies were performed in CXCL10 knockout mice and mice treated with anti-CXCL10 immunoglobulin G (IgG). Endpoints included leukocyte trafficking and activation, core body temperature, plasma cytokine concentrations, bacterial clearance and survival.

**Results:**

CXCL10 was present at high concentrations in plasma and peritoneal cavity during CLP-induced septic shock. Survival was significantly improved in CXCL10 knockout (CXCL10KO) mice and mice treated with anti-CXCL10 IgG compared to controls. CXCL10KO mice and mice treated with anti-CXCL10 IgG showed attenuated hypothermia, lower concentrations of interleukin-6 (IL-6) and macrophage inhibitory protein-2 (MIP-2) in plasma and lessened natural killer (NK) cell activation compared to control mice. Compared to control mice, bacterial burden in blood and lungs was lower in CXCL10-deficient mice but not in mice treated with anti-CXCL10 IgG. Treatment of mice with anti-CXCL10 IgG plus fluids and Primaxin at 2 or 6 hours after CLP significantly improved survival compared to mice treated with non-specific IgG under the same conditions.

**Conclusions:**

CXCL10 plays a role in the pathogenesis of CLP-induced septic shock and could serve as a therapeutic target during the acute phase of septic shock.

## Introduction

The CXC chemokine CXCL10 (also known as interferon-inducible protein 10 (IP-10)) is produced during periods of infection and inflammation in response to type I and type II interferons (IFN) such as IFNα/β and IFNγ, respectively [1-4]. CXCL10 activates the G-protein coupled chemokine receptor CXCR3, an important regulator of natural killer (NK), natural killer T (NKT) and T helper (Th)1 lymphocyte trafficking, in response to viral infections, autoimmune diseases, allotransplantation and cancer [5-10]. Recently, a role for CXCR3 activation in the pathogenesis of severe sepsis has been proposed [11]. Compared to wild-type mice, CXCR3-deficient mice show less systemic cytokine production, attenuated physiologic dysfunction and improved survival during severe sepsis caused by cecal ligation and puncture (CLP) [11]. Large numbers of CXCR3^+^ NK cells migrate from the spleen and blood into the peritoneal cavity during CLP-induced sepsis, a phenomenon that is ablated in CXCR3-deficient mice as well as in mice treated with neutralizing antibodies against CXCR3 [11,12]. Thus, the trafficking of NK cells to the site of infection after CLP parallels the development of systemic inflammation and mortality. Both phenomena are ablated by CXCR3 deficiency or blockade, which raises the contention that the improved outcomes observed in septic mice with CXCR3 deficiency or blockade are due to attenuated trafficking and activation of innate lymphocyte populations. However, further research is needed to determine the mechanisms by which CXCR3 activation facilitates the pathogenesis of septic shock.

High concentrations of CXCL10 are present in peritoneal lavage fluid and plasma during CLP-induced septic shock [11]. The increased concentrations of CXCL10 parallel the trafficking of NK cells into the inflamed and infected peritoneal cavity. Furthermore, high CXCL10 concentrations correlate with the development of physiologic dysfunction and death in the CLP model of sepsis [11]. In clinical studies, plasma CXCL10 concentrations are markedly elevated in septic patients and plasma CXCL10 concentrations correlate with the severity of sepsis in humans [4,13,14]. Punyadeera *et al.*[14] reported that elevated plasma CXCL10 concentrations are predictive of the transition from sepsis to septic shock in critically ill adults. In other studies, elevated plasma CXCL10 concentrations have been shown to be a reliable indicator of sepsis in neonates and infants [15,16]. However, the functional importance of CXCL10 in the pathogenesis of severe sepsis has not been ascertained.

In the present study, the effect of CXCL10 deficiency or blockade on the pathogenesis of CLP-induced septic shock was investigated. Survival, core body temperature, bacterial clearance and systemic cytokine production as well as lymphocyte trafficking and activation were evaluated in CXCL10-deficient mice and in mice treated with neutralizing antibody against CXCL10. CXCL10 blockade was initiated prior to or after the onset of sepsis. In the latter studies, the goal was to determine whether CXCL10 blockade might serve as a therapeutic target during experimental septic shock.

## Methods

### Mice

Female and male, 10- to 12-week-old C57BL/6 J wild-type mice and homozygous CXCL10-null mice (B6.129S4-*Cxcl10*^*tm1Adl*^/J, CXCL10KO) were purchased from the Jackson Laboratory (Bar Harbor, ME, USA). CXCL10KO mice were genotyped at our facility to assure authenticity. All studies were approved by the Institutional Animal Care and Use Committees at the University of Texas Medical Branch and Vanderbilt University Medical Center and complied with the National Institutes of Health Guide for the Care and Use of Experimental Animals, which conforms to internationally recognized guidelines for the ethical treatment of animals.

### Cecal ligation and puncture (CLP)

CLP was performed as previously described [11]. Briefly, mice were anesthetized with 2 to 3% isoflurane in oxygen. After shaving and aseptic preparation of the surgical site, a 1- to 2-cm midline incision was made through the abdominal wall; the cecum was identified and ligated with a 3-0 silk suture at 0.5 to 1 cm from the tip, depending on the desired severity of the model. A double puncture of the cecal wall was performed with a 20-gauge needle. The incision was closed with 3-0 Prolene suture. Mice were resuscitated with intraperitoneal injection of 1 ml of lactated Ringer’s (LR) solution, with or without primaxin (25 mg/kg, Merck & Co, Whitehouse Station, NJ, USA), immediately after CLP. Primaxin is a formulation of imipenem (a potent, broad-spectrum thienamycin antibiotic) and cilastatin sodium (the inhibitor of the renal dipeptidase, dehydropeptidase I). Mice received buprenorphene (0.1 mg/kg) subcutaneously at 30 minutes prior to CLP and twice daily thereafter for analgesia.

### Experimental protocols

#### ***Anti-CXCL10 pretreatment experiments***

Mice received intraperitoneal injection with polyclonal anti-CXCL10 IgG or nonspecific IgG (100 μg, R&D Systems, Minneapolis, MN, USA) at 1 hour before CLP. Mice were resuscitated with lactated Ringer’s (LR) solution containing primaxin immediately after CLP. Subsets of mice were monitored for survival. In additional mice, rectal temperature was measured and samples were harvested for measurement of tissue and blood bacterial burden and cytokine concentrations.

#### ***Anti-CXCL10 post-treatment experiments***

Mice underwent CLP and received intravenous treatment with polyclonal anti-CXCL10 IgG or non-specific IgG (100 μg in 0.2 ml LR) at 2 or 6 hours after CLP. At the time of IgG treatment, mice also received intraperitoneal injection with LR plus primaxin. Antibodies were given intravenously in the post-treatment experiments to speed systemic dissemination. Mice were monitored for survival for 7 days after CLP.

#### ***Recombinant CXCL10 treatment experiments***

Mice underwent CLP and were treated with intravenous (IV) recombinant mouse CXCL10 (R&D Systems) at 1 ug/mouse, or vehicle at 8 hours after CLP. Mice were monitored for survival and rectal temperature was measured at the indicated time points. Mice received intraperitoneal resuscitation with LR immediately after CLP.

### Flow cytometry

Splenic and intraperitoneal leukocytes were harvested as previously described [11,17]. Measurements of NK cell numbers were made at 6 hours after CLP in mice that did not receive antibiotic treatment. Our previous studies show significant NK cell migration into the peritoneal cavity at 6 to 8 hours after CLP [11]. Briefly, spleens were harvested and placed in 35-mm dishes containing RPMI-1640 media with 10% fetal bovine serum, and mashed with the plunger from a 1-ml syringe. The splenocyte suspensions were passed through a 70-μm nylon mesh and erythrocytes were lysed (Erythrocyte Lysis Kit; R&D Systems). Isolated splenocytes were resuspended (1 × 10^7^ cells/ml) in PBS. Intraperitoneal leukocytes were harvested by lavage with 3 ml of PBS. After washing and counting, cells were resuspended in PBS (1 × 10^7^ cells/ml). Cell viability was greater than 95% in all cases as determined by trypan blue exclusion.

Isolated leukocytes (1 × 10^6^/tube) were placed in polystyrene tubes and incubated with anti-mouse CD16/32 (eBioscience, San Diego, CA, USA) to block non-specific Fc receptor-mediated antibody binding. Fluorochrome-conjugated antibodies or isotype controls (0.5 to 1 g/tube) were added, incubated (4°C) for 30 minutes, and washed with 2 ml of cold PBS. Cells were fixed with 250 μl of 1% paraformaldehyde. Antibodies used in the analyses included anti-CD3, anti-NK1.1, anti-CD11b and anti-CD27 (eBioScience) as well as appropriate isotype control antibodies. Samples were analyzed with an Accuri C6 flow cytometer (BD Biosciences). Data were analyzed using Accuri C6 software.

### Cytokine measurements

Heparinized blood was obtained by carotid laceration and plasma was harvested following centrifugation (2000 × g for 15 minutes). The peritoneal cavity was lavaged with 3 ml of sterile PBS. Urea was used as a standard for dilution of peritoneal lavage samples because urea readily diffuses freely throughout the tissue and fluid compartments. When the urea concentrations in plasma and a peritoneal lavage sample are known, the dilution of the initial volume of peritoneal fluid obtained can be calculated as previously described [18,19]. The urea content of peritoneal lavage and plasma samples were determined using iStat Chem8 cartridges (Abbott Point of Care, Princeton, NJ, USA). CXCL10, IL-6 and macrophage inhibitory protein-2 (MIP-2) concentrations were measured using ELISA according to the manufacturer’s protocols (eBioscience). Cytokine concentrations were determined by measuring optical density at 450 nm using a microtiter plate reader (Dynatech Laboratories, Chantilly, VA, USA). Previous studies from our laboratory, and others, show that the magnitude of systemic IL-6 and MIP-2 production during CLP-induced sepsis parallels the severity of physiologic dysfunction and mortality [11,20]. Thus, those cytokines were used as markers of systemic inflammation.

### Measurement of temperature and bacterial counts

Body temperature was measured by insertion of a lubricated rectal temperature probe prior to and at various times after CLP. Previous studies show that the magnitude of hypothermia correlates with mortality in the CLP model [11]. Thus, rectal temperature was used as a marker of physiological dysfunction in our model. Arterial blood gas analysis was performed on heparinized blood using CG4+ iStat Cartridges (Abbott Diagnostics). Bacterial counts were performed on blood, peritoneal lavage fluid and lung homogenates. Samples of blood and peritoneal lavage fluid were obtained as described above. Lung tissue was harvested under aseptic conditions, weighed and homogenized in sterile PBS to achieve a final concentration of 11 mg of tissue per ml of saline. Samples were serially diluted in sterile saline and plated on tryptic soy agar. Plates were incubated (37°C) for 24 to 48 hours and colony counts were performed by direct visualization.

### Statistics

All data were analyzed using GraphPad Prism software (GraphPad Software, San Diego, CA, USA). Data from multiple group experiments were analyzed using one-way analysis of variance (ANOVA) followed post hoc by the Tukey multi-comparison test. Comparison of data from experiments using two groups of subjects was achieved using the unpaired *t*-test. For measurements of bacterial colony-forming units (CFU), groups were compared using the nonparametric Kruskal-Wallis test, followed post hoc by Dunn’s test. Survival data were analyzed using the log rank test. A value of *P* <0.05 was considered statistically significant for all experiments. All values are presented as the mean ± standard error of the mean (SEM), except for bacterial counts, for which median values are designated.

## Results

### CXCL10 production during CLP-induced sepsis

**Figure 1:**
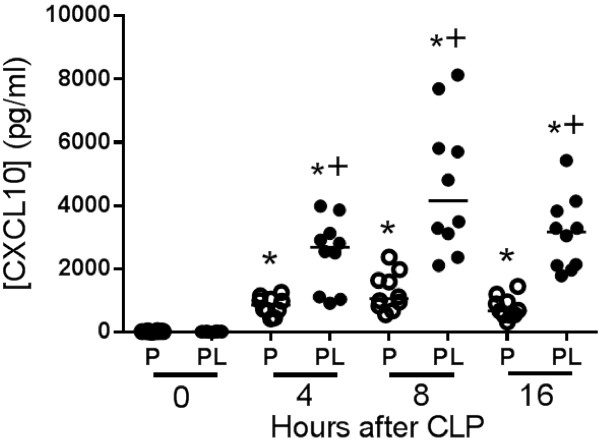
**CXC chemokine 10 (CXCL10) concentrations in plasma and peritoneal lavage during cecal ligation and puncture (CLP)-induced sepsis*. ***Plasma and peritoneal lavage were harvested at the indicated time points after CLP. CXCL10 concentrations were measured by ELISA: n = 10 mice per group: *P <0.05 compared to 0-hour control, ^+^*P* <0.05 compared to plasma.

Concentrations of CXCL10 increased in plasma and the peritoneal cavity within 4 hours after CLP and remained elevated at 8 and 16 hours with the highest concentrations being measured at 8 hours after CLP (Figure 1). CXCL10 concentrations in peritoneal lavage were significantly higher than in plasma at 4, 8 and 16 hours after CLP. CXCL10 was not detectable in plasma or peritoneal lavage fluid in CXCL10 knockout (CXCL10KO) mice (data not shown).

### Effect of CXCL10 deficiency on survival, rectal temperature and plasma cytokine concentrations during CLP-induced sepsis

**Figure 2:**
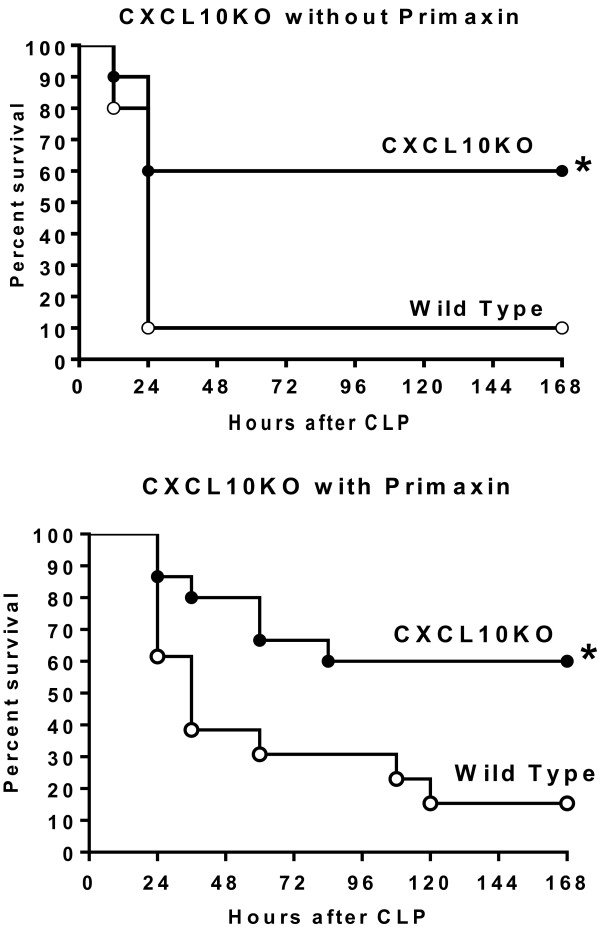
**Survival of wild-type and CXC chemokine 10 (CXCL10)-deficient mice during cecal ligation and puncture (CLP)-induced sepsis*. ***Mice underwent CLP, received intraperitoneal resuscitation with lactated Ringer's solution (LR) (with or without primaxin) and were monitored for survival: n = 10 to 15 mice per group: *P <0.05 compared to wild-type mice.

Survival was measured in control- and CXCL10-deficient mice (Figure 2). In the absence of antibiotic treatment, CXCL10 knock-out (CXCL10KO) mice showed significantly higher survival rates compared to wild-type mice (60% versus 10%). Likewise, CXCL10KO mice resuscitated with fluids and antibiotics at the time of CLP showed significantly higher survival rates compared to wild-type mice (60% versus 13%). Although survival was prolonged in mice receiving antibiotics, overall mortality rates were similar with or without antibiotic treatment.

**Figure 3:**
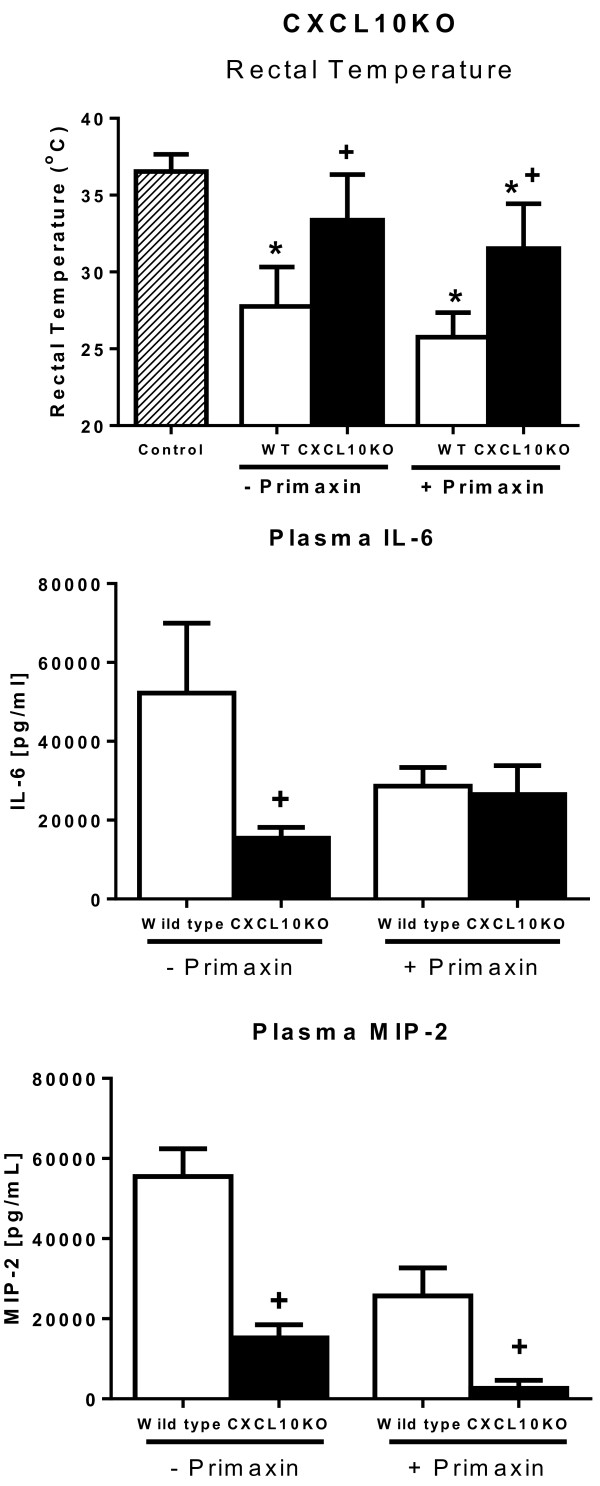
**Effect of CXC chemokine 10 (CXCL10) deficiency on core body temperature and plasma cytokines during cecal ligation and puncture (CLP)-induced sepsis.** Rectal temperature and plasma IL-6 and Macrophage inhibitory protein-2 (MIP-2) concentrations were measured in mice treated with or without primaxin after CLP: n = 10 mice per group; *P <0.05 compared to control, ^+^*P* <0.05 compared to wild-type mice. WT, wild-type. Measurements were made at the onset of mortality in our model: at 24 hours post-CLP in mice that received primaxin and 12 hours post-CLP in mice that did not receive antibiotics.

Hypothermia serves as a reliable indicator of physiological integrity and survival in mice during CLP-induced sepsis [11]. Studies were undertaken to assess rectal temperature in wild-type and CXCL10KO mice during CLP-induced sepsis. In mice that did not receive Primaxin, temperature was measured at 12 hours after CLP, whereas temperature was measured at 24 hours after CLP in mice that received primaxin as part of the resuscitation protocol (Figure 3). Those time points were chosen due to the effect of antibiotic treatment on time to initial mortality as shown in Figure 2. Specifically, initial mortality was observed by 12 hours in mice that did not receive antibiotics, whereas antibiotic treatment prolonged initial mortality until 24 hours after CLP. Thus, measurements were made at the time on mortality onset in the respective groups. In mice that did not receive primaxin treatment, rectal temperature was significantly decreased in wild-type, but not CXCL10KO, mice compared to non-septic controls. Rectal temperature was significantly higher in CXCL10KO mice compared to wild-type mice (Figure 3). Wild-type and CXCL10KO mice treated with Primaxin exhibited significantly decreased rectal temperature at 24 hours after CLP compared to non-septic controls. Rectal temperature was significantly higher in CXCL10KO mice compared to wild-type mice (Figure 3).Plasma IL-6 and MIP-2 concentrations were also measured at 12 or 24 hours after CLP in mice treated without or with primaxin, respectively (Figure 3). In mice that did not receive treatment with primaxin, plasma IL-6 and MIP-2 concentrations were significantly lower in CXCL10KO mice compared to wild-type mice. In mice that received primaxin treatment, plasma MIP-2 but not IL-6 concentrations were lower in CXCL10KO mice compared to wild-type mice.

### Effect of CXCL10 deficiency on bacterial clearance during CLP-induced sepsis

**Figure 4:**
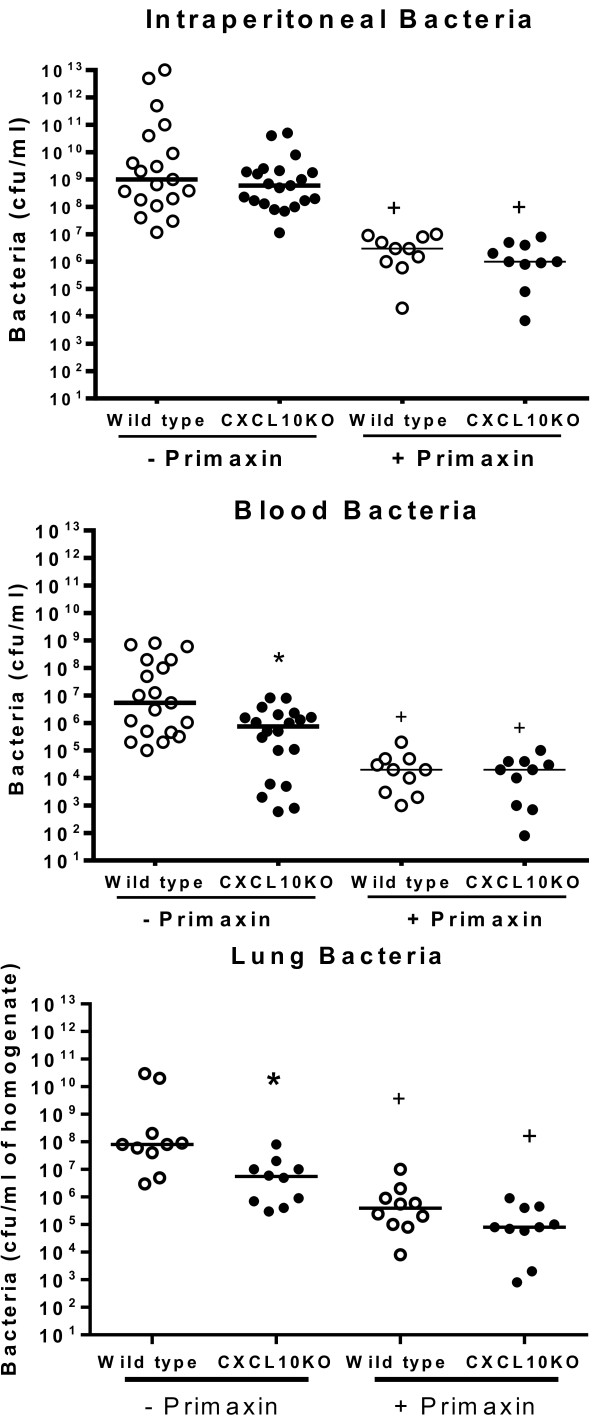
**Effect of CXC chemokine 10 (CXCL10) deficiency on bacterial burden during during cecal ligation and puncture (CLP)-induced sepsis.** Bacterial colony forming units (CFU) were measured in mice treated with or without primaxin after CLP; n = 10 to 20 mice per group; *P <0.05 compared to wild-type, ^+^*P* <0.05 compared to mice that did not receive primaxin treatment. Measurements were made at the onset of mortality in our model: at 24 hours post-CLP in mice that received primaxin and 12 hours post-CLP in mice that did not receive antibiotics.

Bacterial counts were measured in control and CXCL10-deficient mice that were treated with or without Primaxin (Figure 4). Treatment with Primaxin significantly decreased bacterial counts in the peritoneal cavity, blood and lungs by greater than two orders of magnitude compared to the same tissues from mice that did not receive primaxin treatment (Figure 4). Significant differences in intraperitoneal bacterial counts were not observed when comparing CXCL10KO and wild-type mice, regardless of whether primaxin was given (Figure 4). Bacterial counts in blood and lung were significantly lower in CXCL10KO mice than in wild-type mice when primaxin was not given. However, in primaxin-treated mice, no differences in bacterial burden were observed in blood and lungs when comparing wild-type and CXCL10KO mice (Figure 4).

### Effect of CXCL10 blockade on lymphocyte trafficking and activation

**Figure 5:**
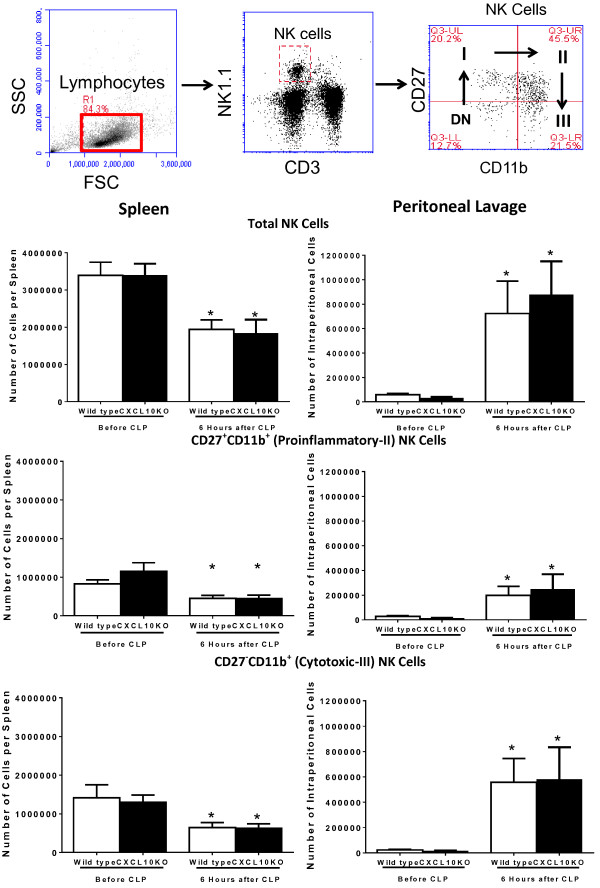
**Natural killer cell trafficking during cecal ligation and puncture (CLP)-induced sepsis in wild-type and CXC chemokine 10 (CXCL10)-deficient mice*. ***Splenocytes and intraperitoneal leukocytes were harvested prior to or 6 hours after CLP. Natural killer (NK) cells were identified using flow cytometry as NK1.1^+^CD3^-^ lymphocytes and were further characterized based on CD11b and CD27 expression. DN, double negative NK cells, I = CD27^+^CD11b^-^ immature NK cells, II = CD27^+^CD11b^+^ pro-inflammatory NK cells, III = CD27^-^CD11b^+^ cytotoxic NK cells; n = 5 to 11 mice per group. *P <0.05 compared to “Before CLP”.

Studies were undertaken to assess lymphocyte trafficking in wild-type and CXCL10KO mice after CLP. All studies were performed in the absence of antibiotic treatment. Natural killer (NK) cells can be subdivided into immature (I, CD27^+^CD11b^-^), pro-inflammatory (II, CD27^+^CD11b^+^) and cytotoxic (III, CD27^-^CD11b^+^) subsets based on CD27 and CD11b expression (Figure 5) [21]. The upper panels of Figure 5 shows the gating strategy used to identify NK cell subsets. Total NK cells were identified as NK1.1^+^CD3^-^ lymphocytes (Figure 5). NK cell subsets were identified as pro-inflammatory or cytotoxic based on CD27 and CD11b expression as described above. A significant decline in total, pro-inflammatory and cytotoxic NK cells was observed in the spleen by 6 hours after CLP and corresponded with an increase in intraperitoneal numbers of the same NK cell subsets over the same time period (Figure 5). The numbers of immature NK cells in spleen and peritoneal lavage did not change during the first 6 hours after CLP (data not shown). No significant difference in splenic or intraperitoneal NK cell numbers was noted when comparing wild-type and CXCL10KO mice (Figure 5). The numbers of T (NK1.1^-^CD3^+^) or NKT (NK1.1^+^CD3^+^) cells in spleen and peritoneal lavage did not change at 6 hours after CLP in either group (data not shown).

**Figure 6:**
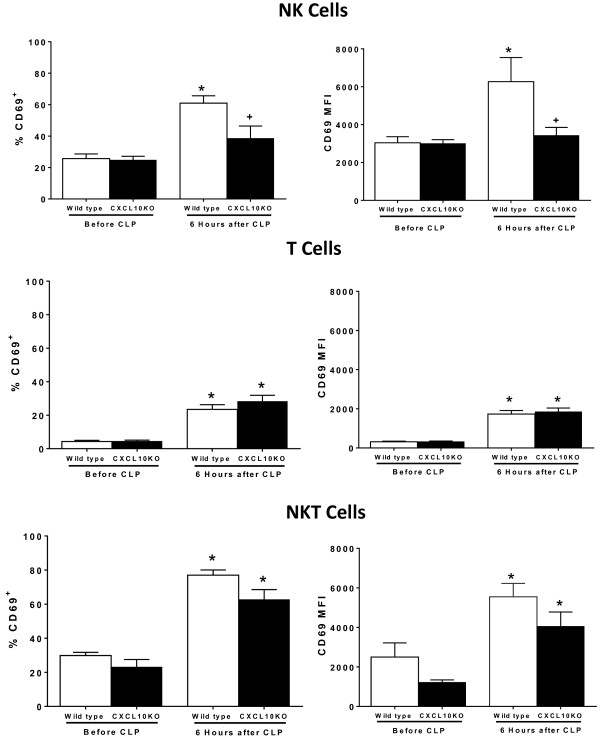
**Lymphocyte activation during cecal ligation and puncture (CLP)-induced sepsis in wild-type and CXC chemokine 10 (CXCL10)-deficient mice*. ***Intraperitoneal leukocytes were harvested prior to or 6 hours after CLP. Surface CD69 expression was measured on natural killer (NK) (NK1.1^+^CD3^-^), NKT (NK1.1^+^CD3^+^) and T (NK1.1^-^CD3^+^) lymphocytes using flow cytometry; n = 5 to 6 mice per group. *P <0.05 compared to “Before CLP”, ^+^*P* <0.05 compared to wild-type.

Further studies were undertaken to assess early activation of intraperitoneal lymphocytes using CD69 as a biomarker (Figure 6). Intraperitoneal NK, T and NKT lymphocytes from wild-type mice showed evidence of activation after CLP as indicated by significantly increased percentages of CD69-positive cells and increased CD69 mean fluorescence intensity (MFI) (Figure 6). At 6 hours post-CLP, the percentage of CD69^+^ NK cells and the CD69 MFI on NK cells were significantly lower in CXCL10KO mice compared to wild-type mice. For T and NKT cells, the percentages of CD69^+^ cells and the CD69 MFI were not significantly different when comparing lymphocytes from wild-type and CXCL10KO mice (Figure 6).

### Effect of CXCL10 blockade on survival, core body temperature, plasma cytokine concentrations and bacterial burden during CLP-induced sepsis

**Figure 7:**
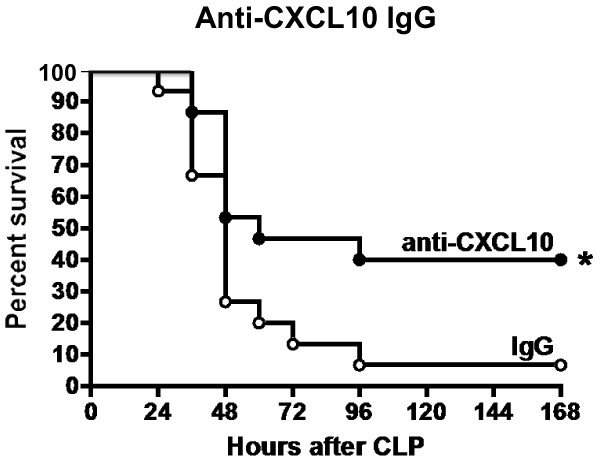
**Effect of CXC chemokine 10 (CXCL10) blockade on survival during cecal ligation and puncture (CLP)-induced sepsis*. ***Mice were treated with non-specific IgG or polyclonal anti-CXCL10 IgG 1 hour prior to CLP. Mice then underwent CLP and were monitored for survival. Mice received intraperitoneal resuscitation with 1 ml of lactated Ringer’s solution with primaxin (25 mg/kg) at the time of CLP; n = 10 to 15 mice per group, *P <0.05 compared to IgG-treated mice.

**Figure 8:**
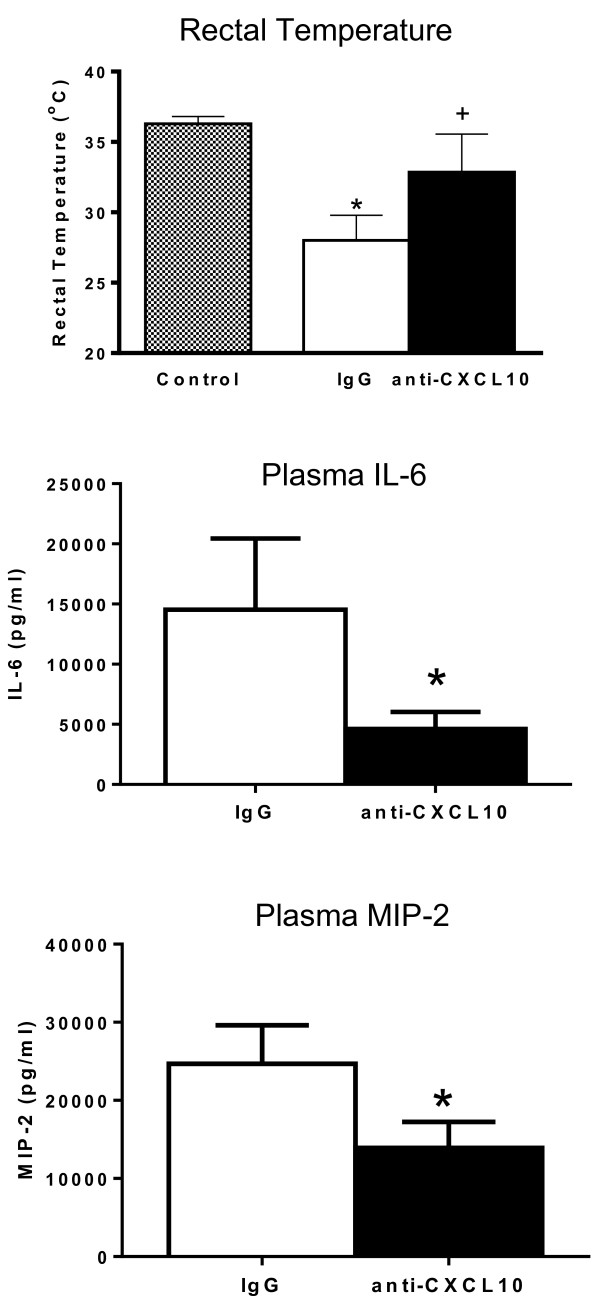
**Effect of CXC chemokine 10 (CXCL10) blockade on rectal temperature and plasma cytokine concentrations during cecal ligation and puncture (CLP)-induced sepsis.** Mice were treated with non-specific IgG (IgG, 100 ug intraperitoneal (IP)) or anti-CXCL10 IgG (anti-CXCL10, 100 ug IP) 1 hour prior to CLP. Mice received IP resuscitation with 1 ml of lactated Ringer’s solution plus primaxin (25 mg/kg) at the time of CLP; n = 10 mice per group, *P <0.05 compared to control, ^+^*P* <0.05 compared to non-specific IgG-treated mice. αCXCL10, anti-CXCL10 IgG. Measurements were made at 24 hours after CLP. MIP-2, Macrophage inhibitory protein-2.

**Figure 9:**
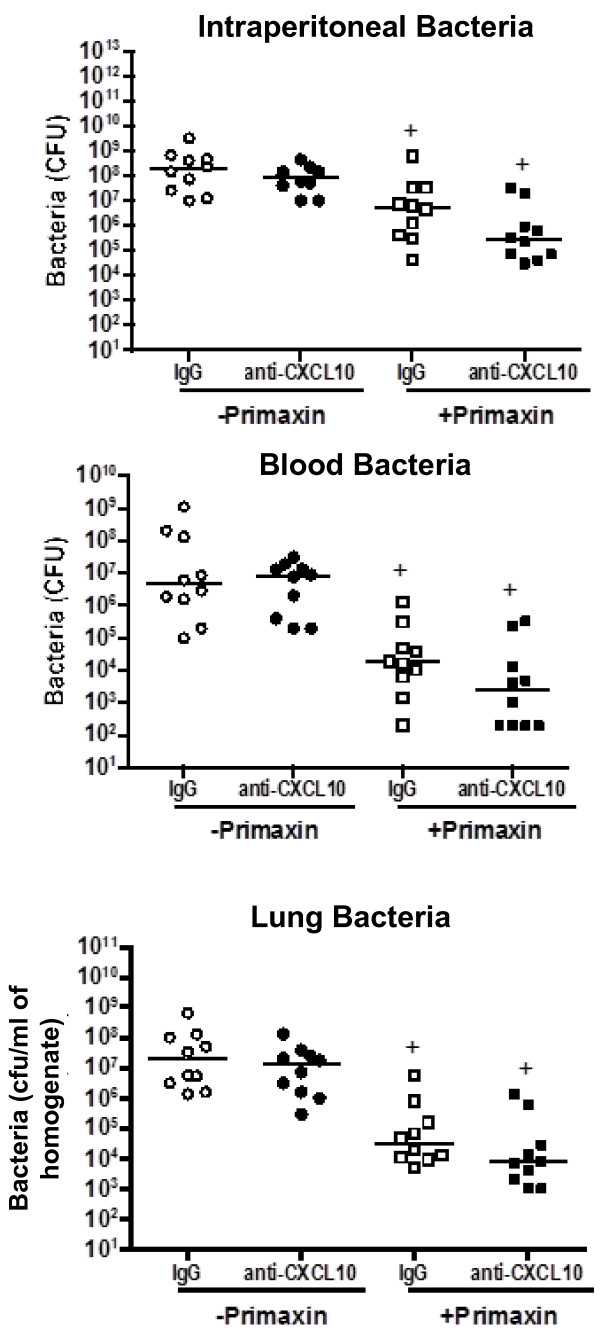
**Effect of CXC chemokine 10 (CXCL10) blockade on bacterial burden during cecal ligation and puncture (CLP)-induced sepsis*. ***Plasma and tissue bacterial counts were measured in mice treated with or without primaxin after CLP. Mice were treated with non-specific IgG (IgG, 100 ug intraperitoneal (IP)) or anti-CXCL10 IgG (anti-CXCL10, 100 ug IP) 1 hour prior to CLP. Mice received intraperitoneal resuscitation with 1 ml of lactated Ringer’s solution, with or without primaxin (25 mg/kg), at the time of CLP; n = 10 mice per group, ^+^*P* <0.05 compared to “- Primaxin” group. Measurements were made at 24 hours after CLP.

Studies were undertaken to determine whether blockade of CXCL10 alters survival during CLP-induced sepsis (Figure 7). Wild-type mice were treated with anti-CXCL10 IgG or non-specific IgG at 1 hour prior to CLP. Mice were resuscitated with LR and primaxin at the time of CLP. Survival was significantly improved in mice treated with anti-CXCL10 IgG compared to mice treated with non-specific IgG (40% versus 7%, Figure 7).In further studies, rectal temperature and plasma IL-6 and MIP-2 concentrations were measured in wild-type mice treated with anti-CXCL10 IgG or non-specific IgG (Figure 8). Mice were resuscitated with LR plus primaxin. CLP induced a significant decrease in temperature in wild-type mice treated with non-specific IgG compared to non-septic controls. Rectal temperature was not significantly decreased in mice treated with anti-CXCL10 IgG compared to non-septic controls and was significantly higher than in mice treated with non-specific IgG (Figure 8). Examination of plasma cytokines showed that IL-6 and MIP-2 concentrations were significantly lower in the anti-CXCL10 IgG group compared to the non-specific IgG group (Figure 8).Bacterial counts were measured in mice that were treated with or without primaxin (Figure 9). Bacterial counts in peritoneal lavage fluid, blood and lungs were not significantly different between mice treated with non-specific IgG or anti-CXCL10 IgG, regardless of whether mice were treated with primaxin (Figure 9). Bacterial counts at all measured sites were significantly lower in mice treated with primaxin compared to those that did not receive primaxin treatment (Figure 9).

### Effect of anti-CXCL10 IgG treatment on survival when administered after the onset of sepsis

**Figure 10:**
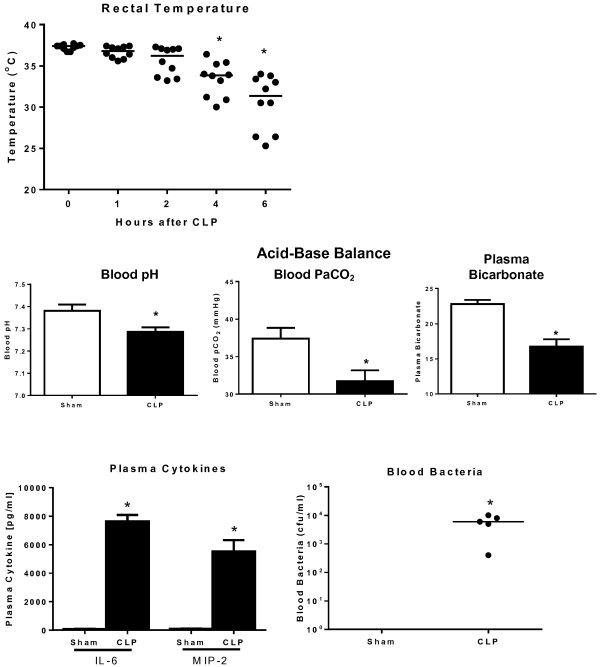
**Progression of sepsis in the cecal ligation and puncture (CLP) model*. ***Mice underwent sham or CLP procedures and rectal temperature was measured at the indicated time points. At 6 hours after CLP blood was harvested for measurement of plasma cytokines, acid-base balance and bacterial burden; n = 5 to 10 mice per group, *P <0.05 compared to 0 hour (no CLP) control or sham. MIP-2, Macrophage inhibitory protein-2.

**Figure 11:**
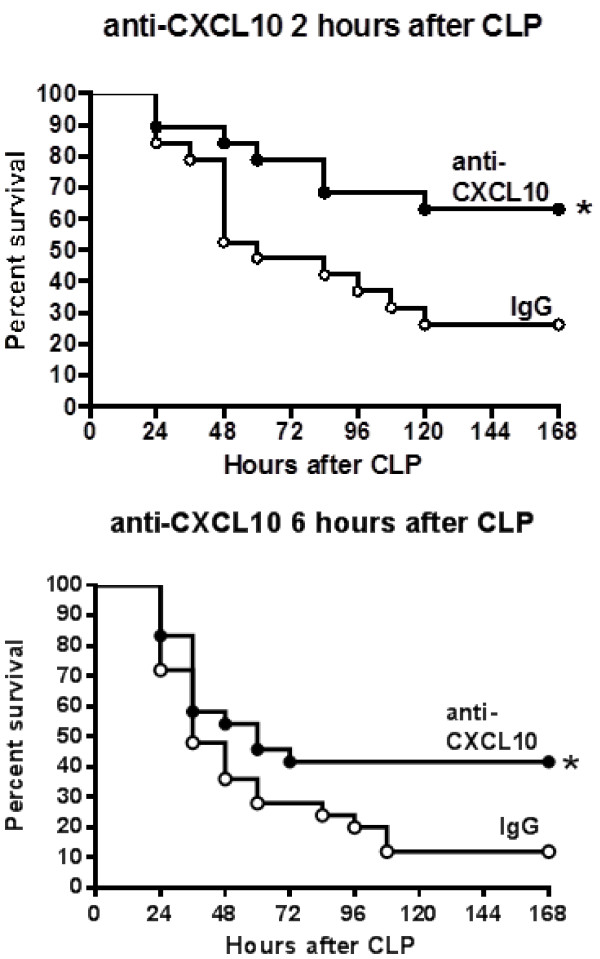
**CXC chemokine 10 (CXCL10) blockade improves survival when initiated after cecal ligation and puncture (CLP)*CPS. ***Mice underwent CLP and were monitored for survival. Mice were treated with anti-CXCL10 IgG (anti-CXCL10, 100 μg intravenously (IV)) or non-specific IgG (IgG, 100 μg IV) at 2 or 6 hours after CLP. Mice received intraperitoneal resuscitation with 1 ml of lactated Ringer’s solution and primaxin (25 mg/kg) at the time of IgG treatment; n = 20 (2 hours post-treatment) to 25 (6 hours post-treatment) mice per group; *P <0.05 compared to mice treated with non-specific IgG.

Studies were undertaken to assess whether initiation of CXCL10 blockade after the onset of sepsis would improve survival. Initial studies were undertaken to characterize the progression of sepsis in our model and determine whether evidence of sepsis was present at the time of anti-CXCL10 IgG treatment (Figure 10). Rectal temperature was measured at the indicated time points and showed a significant decline at 4 hours after CLP and a further decline at 6 hours after CLP compared to non-septic (0 hours) mice (Figure 10). Assessment of acid-base balance at 6 hours after CLP showed metabolic acidosis compared to sham mice, characterized by a significant decrease in blood pH and bicarbonate concentrations (Figure 10). P_a_CO_2_ was also significantly lower in septic mice compared to sham controls, reflecting respiratory compensation for the emerging metabolic acidosis (Figure 10). Plasma concentrations of IL-6 and MIP-2 were significantly increased at 6 hours after CLP and mice were also bacteremic at that time point as indicated by positive blood cultures in all mice (Figure 10).Mice were treated with anti-CXCL10 IgG or non-specific IgG in combination with fluids and primaxin at 2 or 6 hours after the CLP procedure (Figure 11). Survival was significantly improved in wild-type mice treated with anti-CXCL10 IgG at 2 hours (65% versus 25%) and 6 hours after CLP (40% versus 15%) compared to mice treated with non-specific IgG (Figure 11).

## Discussion

The present study shows that CXCL10 deficiency or blockade confers protection from CLP-induced septic shock in mice, regardless of whether antibiotics are given. Sepsis-induced hypothermia and systemic cytokine production were attenuated and survival rates were higher in CXCL10-deficient mice and in mice treated with a neutralizing antibody against CXCL10, compared to control mice. Collectively, those observations support the contention that CXCL10 plays a functional role in facilitating inflammation and physiology during CLP-induced acute septic shock. In addition, CXCL10 blockade could serve as a therapeutic target during acute septic shock, as treatment with anti-CXCL10 IgG and antibiotics was effective in improving survival when administered at 2 or 6 hours after the onset of sepsis. The improvement in survival observed when mice were treated with anti-CXCL10 IgG at 6 hours after CLP was particularly compelling because mice showed clinical signs of sepsis such as hypothermia, metabolic acidosis, systemic inflammation and bacteremia at the time of treatment.

CXCL10 has been shown to serve as a useful biomarker in human sepsis. Several studies have reported increased concentrations of CXCL10 in the plasma of septic human adults, infants and neonates [4,13,15,16]. Punyadeera and colleagues showed that high plasma CXCL10 concentrations are associated with the transition from sepsis to septic shock in critically ill adult patients [14]. Elevated plasma CXCL10 concentrations have also been shown to be a reliable indicator of sepsis in neonates and infants [15,16]. However, the functional importance of CXCL10 in facilitating the pathogenesis of severe bacterial sepsis had not been directly examined. Yet, prior studies have shown that CXCL10 contributes to acute organ injury and physiologic dysfunction in other acute inflammatory syndromes. Ichikawa and colleagues showed that mice lacking CXCL10 are resistant to acute lung injury caused by acid aspiration or influenza virus infection [22]. CXCL10 neutralization has been effective in attenuating NK and CD8 T cell-mediated hepatocellular injury during fulminant Dengue virus infection [23]. A role for CXCL10 in the pathogenesis of cerebral malaria has also been described [24,25]. Other studies have indirectly supported a role for CXCL10 in the pathogenesis of septic shock. Seyoum *et al.* reported that virulent strains of *S. pneumoniae* induce high CXCL10 expression and that CXCR3-deficient mice exhibit attenuated pulmonary inflammation and improved outcomes during sepsis caused by *Streptococcal* pneumonia [26]. In further studies, Martin and colleagues showed that virulent strains of methicillin-resistant *Staphylococus aureus* (MRSA) induced high concentrations CXCL10 in the lung after intrapulmonary challenge and that CXCR3 neutralization decreases intrapulmonary inflammation [27]. The present studies show that CXCL10-deficient mice are resistant to CLP-induced septic shock and more directly support a cause-and-effect relationship between CXCL10 and the pathogenesis of sepsis.

The production of CXCL10 is largely regulated by activation of type I and type II IFN receptors by IFNα/β and IFNγ, respectively [28,29]. The transcription factor STAT-1 is an important mediator for IFN-induced gene transcription and plays a central role in regulating CXCL10 expression [29-31]. Previous studies from our laboratory show that STAT1-deficient mice are resistant to CLP-induced septic shock [32]. STAT1 knockout mice exhibited improved survival, attenuated systemic inflammation and diminished CXCL10 production during CLP-induced sepsis. In additional studies, decreased systemic inflammation and improved survival have been demonstrated in type 1 IFN receptor- and IFNβ-deficient mice during fulminant CLP-induced peritonitis and endotoxin-induced shock, respectively [33-35]. Dejager *et al.* reported that blockade of type I IFN signaling was protective in models of endotoxin and CLP-induced severe sepsis [35]. In other studies, Lachance and colleagues showed that IFNγ-deficient mice are resistant to septic shock caused by a virulent strain of *Streptococcus suis*[36]. That finding is consistent with previous studies that demonstrated attenuated hyperinflammation and improved survival in IFNγ-deficient mice in models of CLP- and endotoxin-induced shock [37,38]. Taken together, those prior findings and the results from the present study support a role of IFNα/β, IFNγ and CXCL10 in the pathogenesis of septic shock.

As with other mediators of hyperinflammation and shock during systemic bacterial infections, it is likely that CXCL10 as well as IFNα/β and IFNγ play beneficial roles in the containment of local and less severe systemic infections. Kelly-Scumpia and colleagues reported that type I IFN receptor-deficient mice have increased susceptibility to lower severity CLP-induced sepsis in which control mice showed mortality rates of less than 10% [39]. The heightened susceptibility was associated with impaired bacterial clearance. They further reported that the vulnerability can be reversed by treatment with CXCL10 and concluded that CXCL10 is an important regulator of antimicrobial function during low-severity CLP-induced sepsis in adult mice. Reim *et al.* also reported that IFN-inducible responses, including production of CXCL10, are beneficial for facilitating bacterial clearance mechanisms in a lower-severity model of CLP-induced sepsis [40]. In further studies, Cuenca *et al.* showed that CXCL10 is important for neutrophil and macrophage recruitment during neonatal sepsis [41]. In those studies, blockade of CXCL10 decreased myeloid cell recruitment to the site of infection, decreased bacterial clearance and increased mortality. Likewise, neonatal CXCR3KO mice showed decreased numbers of neutrophils and macrophages at the site of infection and increased mortality compared to wild-type controls. The investigators postulated that CXCL10 regulates beneficial myeloid cell recruitment during bacterial sepsis in neonatal mice. Interestingly, significantly decreased bacterial counts were observed in the blood and lungs, but not peritoneal cavity, of CXCL10 knockout mice in our study. However, differences in bacterial burden were not observed at any of the sites tested in mice treated with anti-CXCL10. Those findings indicate that CXCL10 could have a small inhibitory effect on bacterial clearance during severe sepsis. *In toto*, these combined findings indicate that CXCL10 production can be beneficial or deleterious depending on the severity of sepsis.

The mechanisms by which CXCL10 facilitates systemic inflammation and physiologic dysfunction during severe sepsis remain to be fully ascertained. CXCL10 activates the chemokine receptor CXCR3 and is an important regulator of lymphocyte trafficking in a variety of diseases characterized by infection or inflammation [1,2,11]. CXCR3 is expressed primarily by NK, NKT and T lymphocytes, all of which have been implicated in the pathogenesis of septic shock [1]. Previous studies from our laboratory and from others have demonstrated that NK and CD8^+^ T cells contribute to systemic inflammation and physiologic dysfunction during sepsis or polytrauma [17,42-44]. Hu and colleagues reported that NKT cells play a functional role in the pathogenesis of CLP-induced septic shock while other investigators have shown that NKT cells facilitate systemic inflammation during lethal challenge with lipopolysaccharide (LPS) [45,46]. Evidence indicates that CXCR3^+^ NK cells migrate into the inflamed peritoneal cavity from blood and spleen early during the course of CLP-induced sepsis. In addition, CXCR3-mediated NK cell trafficking parallels the development of physiologic dysfunction and systemic inflammation during experimental septic shock [11,17]. In the present study, we observed trafficking of NK cells from spleen to peritoneal cavity within 6 hours after CLP and extended our previous findings by showing the migration of both the CD27^+^CD11b^+^ pro-inflammatory and CD27^-^CD11b^+^ cytotoxic NK cell subsets. Yet, differences in NK cell trafficking were not observed when comparing wild-type and CXCL10-deficient mice. That finding indicates that CXCL10 alone is not responsible for NK cell trafficking during CLP-induced sepsis, which is not surprising, given the redundancy of CXCR3 ligands [1]. Our previous studies show that high levels of the CXCR3 ligands CXCL9 and CXCL10 are produced after CLP and that a concentration gradient exists such that concentrations of each cytokine are higher in the peritoneal cavity than in plasma [11]. Thus, it is likely that CXCL9 also contributes to CXCR3-mediated NK cell trafficking and serves as an important NK cell chemoattractant in the absence of CXCL10. Further studies are required to fully define the chemokine milieu that mediates NK cell trafficking during sepsis.

Although CXCL10-mediated NK cell trafficking does not appear to be a crucial factor in facilitating the development of shock in the CLP model, CXCL10-induced NK cell activation may play a role. NK cell activation and systemic inflammation were attenuated in CXCL10-deficient mice as indicated by lessened expression of the early activation marker CD69 by intraperitoneal NK cells and decreased systemic cytokine production. This raises the possibility that CXCL10 serves as an activating factor for NK cells, independent of its role as a chemotactic agent. Previous studies have shown that CXCL10 can facilitate systemic inflammation during acute inflammatory syndromes [22,47]. However, further work is needed to determine how CXCL10 facilitates the pro-inflammatory functions of innate lymphocytes during severe sepsis.

It appears that CXCL10 has the potential to serve as a therapeutic target during acute septic shock. In the present study, blockade of CXCL10, in combination with antibiotic treatment at 6 hours after CLP was effective in improving survival compared to mice treated with non-specific IgG and antibiotics. Our assessment indicates that mice show signs of sepsis at 6 hours after CLP as indicated by the presence of hypothermia, metabolic acidosis and bacteremia. Further work is needed to fully define the potential application of CXCL10 blockade as a therapeutic agent during the acute phase of septic shock. Treatment with IgG molecules, as used in this study, is an attractive approach to altering host immune responses in numerous diseases with immunological or inflammatory etiologies [48,49]. Based on our current observations, immunoglobulin-based strategies appear to represent an attractive strategy for blocking CXCL10 in the setting of severe sepsis. However, there are potential drawbacks to immunoglobulin-based treatment approaches, the most important of which is the persistence of immunoglobulins in the circulation after systemic administration. Long-lived blockade of CXCL10 function could be deleterious to mounting effective downstream anti-microbial responses in patients with sepsis as demonstrated by the studies of Kelly-Scumpia and colleagues [39]. An alternative approach is the use of short-acting small molecular weight inhibitors to block CXCL10 or its receptor, CXCR3. AMG487 is a potent and selective non-competitive CXCR3 inhibitor that has been widely tested in humans [50]. AMG487 undergoes hepatic metabolism and exhibits linear pharmacokinetics during the first 7 days of administration with a half-life of 9.7 hours [51]. Thus, short-acting inhibitors of the CXCL10-CXCR3 axis could serve as attractive agents to apply during the acute phase of sepsis.

## Conclusions

This report shows that CXCL10 deficiency or blockade improves survival, decreases physiologic dysfunction and attenuates systemic cytokine production in mice with septic shock. In addition, the benefits of CXCL10 blockade were evident when anti-CXCL10 IgG was administered at 6 hours after the initiation of sepsis. Our current findings suggest that CXCL10 may contribute to the activation of innate lymphocyte populations, particularly NK cells, during acute septic shock. Further research is needed to fully define the mechanisms by which CXCL10 facilitates the pathogenesis of septic shock and to define the potential of CXCL10 blockade as a therapeutic intervention.

## Key messages

• High levels of CXL10 are produced during experimental and clinical sepsis.

• CXCL10 deficiency or blockade attenuates systemic cytokine production, lessens physiologic dysfunction and improves survival during experimental septic shock.

• CXCL10 contributes to the activation of NK cells during the acute phase of sepsis but does not act alone to facilitate NK cell trafficking.

• CXCL10 could serve as a therapeutic target during the acute phase of sepsis.

## Abbreviations

CD: cluster of differentiation; CFU: colony-forming units; CLP: cecal ligation and puncture; CXCL10: CXC chemokine 10; CXCL9: CXC chemokine 9; CXCR3: CXC chemokine receptor 3; ELISA: enzyme-linked immunosorbent assay; IFN: interferon; IgG: immunoglobulin G; IL-6: interleukin-6; IP-10: interferon-inducible protein 10; LR: lactated Ringer's solution; MFI: mean fluorescence intensity; MIP-2: macrophage inhibitory protein-2; NK: natural killer; NKT: natural killer T; PBS: phosphate-buffered saline; IV: intravenous.

## Competing interests

The authors declare that they have no competing interest.

## Authors’ contributions

DH designed and performed survival studies, cytokine, bacterial burden and temperature measurements and contributed to manuscript preparation. LL designed and performed survival studies, cytokine, bacterial burden and temperature measurements, flow cytometry studies and contributed to manuscript preparation. JB performed cytokine measurements and contributed to manuscript preparation. TT-K contributed to experimental design, data interpretation and manuscript preparation. YG performed flow cytometry experiments and contributed to manuscript preparation. ES oversaw all aspects of the study and contributed to study design, data interpretation and manuscript preparation. All authors have read and approved the manuscript.
